# Defatting of acetone leaf extract of *Acacia karroo* (Hayne) enhances its hypoglycaemic potential

**DOI:** 10.1186/s12906-017-1987-6

**Published:** 2017-10-23

**Authors:** Idris Njanje, Victor P. Bagla, Brian K. Beseni, Vusi Mbazima, Kgomotso W. Lebogo, Leseilane Mampuru, Matlou P. Mokgotho

**Affiliations:** Department of Biochemistry, Microbiology and Biotechnology, Faculty of Science and Agriculture, University of Limpopo, Turfloop Campus, Private Bag X1106, Sovenga, Limpopo 0727 South Africa

**Keywords:** *Acacia karroo*, Diabetes, Enzyme inhibition, Glucose uptake, GLUT4, MAPKs

## Abstract

**Background:**

Conventional drugs used to treat diabetes are too expensive, toxic and rarely available to rural communities. This study was aimed at investigating the phytochemical differences and hypoglycaemic effects (α-amylase enzyme inhibition, glucose uptake, GLUT4 translocation and phosphorylation of MAPKs) of non-defatted and defatted acetone leaf extract of *Acacia karroo.*

**Methods:**

Qualitative phytochemical analyses of extracts were determined using standard chemical tests and total phenolic contents using the Folin-Ciocalteu reagent method. Presence of antioxidant constituents was determined using DPPH scavenging and ferric reducing power assays. Alpha amylase enzyme inhibitory potential was determined chromogenically and cytotoxicity of the extracts on C2C12 muscle and 3T3-L1 cells using the MTT assay. Glucose uptake by the cells was determined colorimetrically and the most active extract was evaluated for its ability to translocate GLUT4 and MAPKs phosphorylation potential using immunofluorescence microscopy and dot blot analysis, respectively.

**Results:**

Phenols, flavonoids, tannins, saponins and cardiac glycosides were detected in both extracts. Defatting of the plant material resulted in low amounts of phenols (0.432 ± 0.014 TAE/mg), DPPH scavenging activity (EC_50_ 0.40 ± 0.012 mg/ml), low toxicity and high ferric reducing power (EC_50_ 1.13 ± 0.017 mg/ml), α-amylase enzyme inhibition (IC_50_ 30.2 ± 3.037 μg/ml) and glucose uptake by both cells. The defatted extract showed an increase in GLUT4 translocation (at 25 μg/ml) with decrease in Akt expression while in combination with insulin showed a decrease in GLUT4 translocation. A finding, that is implicative that the effect of the extract on GLUT4 translocation in C2C12 cells was not Akt dependent. The defatted extract in the absence and presence of insulin show varying phosphorylation levels of CREB, p38, GSK-3 and ERK2 which are important in cell survival and metabolism.

**Conclusion:**

This study represents the first report on the hypoglycemic potential of *A. karroo* and presence of compounds that can be exploited in the search for therapeutics with antidiabetic effect.

## Background

Diabetes mellitus is one of the major metabolic conditions with leading morbidity and mortality rate, which results in macrovascular and microvascular complications caused due to persistent glucose toxicity [1]. It is now said to have reached epidemic status and the figure of people living with diabetes is projected to increase to 522 million globally by the year 2030 [2]. Despite having standard drugs for the management of diabetes mellitus, it continues to be a challenge because so far, there are no reports of drugs that are capable of curing the condition. Standard drugs available have been associated with undesirable effects, in addition to their inaccessibility [3, 4]; meaning that financially challenged people, especially from developing and underdeveloped countries such as most African countries, including South Africa, cannot access these medications [2, 5]. Because of historical recognition [6] and economic pressure, people have resorted to the use of medicinal plants as a conventional way to treat diabetes and its complications. Although there are no reports that one completely recovered from diabetes mellitus because of using medicinal plants, there has been a surge in trying to isolate compounds of therapeutic interest from plants. Reduction of postprandial glucose levels is very important in the management of diabetes mellitus, and plants provide such effects which are similar to available therapies with less to no side effects [7, 8]. Nonetheless, their modes of action are poorly explored.

*Acacia karroo*, commonly known as sweet thorn (English), belongs to the family Fabaceae. The plant is common to the southern Africa region and is widely distributed throughout South Africa, Zambia, Zimbabwe and Angola [9]. The plant is very easy to identify due to its characteristic white paired thorns, brilliant yellow flowers and red like coffee colored bark [10]. The name of the plant has recently been changed to *Vachellia karroo* [11]. The gum produced by *A. karroo* is used against oral thrush and can also be harvested for food during hard times. The Shona people of Zimbabwe use the roots against conditions such as convulsions, gonorrhea and dizziness while the Ndebele people use them for general body pains. Herbivores enjoy a great deal of nutritious forage from *A. karroo*, especially during dry seasons when the grass is dried out. *Acacia karroo* is also effective against fever, malaria, cholera, diarrhoea, dysentery and high blood pressure [12–14]. *Acacia karroo*, just like *Acacia nilotica* contains compounds with hypoglycaemic effects; however, there are no reports that indicate that *A. karroo* induces hypoglycaemic effects. The ability of this plant to exhibit hypotensive effects and interact with biomolecules resulting in inhibitory effects against digestive enzymes [14] prompted us to examine its potential to promote hypoglycemic effects. The aim of this study was to determine the phytochemical, antioxidant, and possible hypoglycemic effects (alpha amylase enzyme inhibition and increased glucose uptake) of the defatted and non-defatted extracts of *A. karroo* and possible modes of action (GLUT4 translocation and phosphorylation of MAPKs) of the most active extract on cells with high glucose uptake.

## Methods

### Plant collection and preparation

Fresh leaves of *Acacia karroo* were collected from University of Limpopo and authenticated at the Larry Leach Herbarium, the plant was assigned a specimen number (UNIN: 121,020) and deposited in the herbarium. Collected leaves were dried at room temperature, protected from light and thereafter ground to a fine powder using a commercial electric blender. Two types of extracts were obtained from the dried leaf material namely, the defatted and non-defatted acetone extracts. For defatting, each plant material (5 g) was extracted (defatted) using hexane (Sigma-Aldrich, S.A) overnight, followed by extraction with acetone (Sigma-Aldrich, S.A). The non-defatted extracts were extracted using acetone (Sigma-Aldrich, S.A) only. The supernatants of each plant material were filtered using a Whatman no.1 filter paper into pre-weighed vials and the filtrates were dried under a stream of air.

### Phytochemical screening

Qualitative tests for various secondary metabolites were conducted using published methods [15, 16].

### Total phenolic content

The quantity of phenols present in each plant extract was determined by the Folin-Ciocalteu reagent method [17]. One milligram per milliliter (1 mg/ml) of each plant extract (0.1 ml) was diluted with 0.4 ml of distilled water, followed by addition of 0.25 ml of Folin reagent. To stop the reaction, 1.25 ml of sodium carbonate (NaCO_3_) was added and the mixture incubated at room temperature in the dark for 30 min, after which absorbance was taken at 725 nm. The blank was prepared by adding all the reagents excluding the plant extracts. The amounts of phenols were determined using the tannic acid standard curve and were expressed as TAE/mg.

### Antioxidant activity

#### DPPH free radical scavenging activity

The antioxidant potential of the extracts was determined by the 2, 2-diphenyl-1-picrylhydrazyl (DPPH) free radical scavenging activity assay [18]. The extracts were serially diluted with distilled water in a 96 well plate, such that the final volume of each plant extract was 100 μl. One hundred microliter (100 μl) of 0.2% DPPH solution was added to each well containing 100 μl of each plant extract at different concentrations. Methanol was used as a blank and DPPH solution was used as a standard control. The 96 well plates were then incubated in the dark for 20 min after which the absorbance was measured at 560 nm using a microtiter plate reader (Promega, U.S.A). The ability of ascorbic acid to scavenge free radicals was used as a standard from which SC_50_ of each extract was compared.

### Ferric reducing power

The ability of the extract to reduce ferric ions to a ferrous complex was determined using the ferric reducing power assay [19]. Different concentrations of each extract (100 μl) were mixed with 250 μl of phosphate buffer (0.2 M, pH 6.6) and 250 μl of Potassium fericcyanide and then incubated at 50 °C for 20 min. To stop the reactions, 250 μl of trichloroacetic acid (TCA) was added and the mixtures centrifuged at 3000 rpm for 10 min. The supernatants of each mixture (250 μl) were aspirated and mixed with 500 μl of deionized water into new aliquots; this was followed by addition of 50 μl of freshly prepared ferric chloride solution. The absorbance of each sample was then read at 700 nm. A blank was prepared using all the reagents except for the plant extract. Ascorbic acid (EC_50_ in mg/ml) was used as a standard to which extracts were compared.

### Pancreatic α-amylase enzyme inhibition

The partial inhibition of pancreatic α-amylase was determined using the chromogenic method adapted by Sigma-Aldrich with minor modifications [20]. Serial dilution of each plant extract (40 μl plant and 160 μl distilled water) reconstituted in DMSO was pre-incubated with 200 μl (4 U/ml) of pancreatic α-amylase (Sigma-Aldrich) (dissolved in ice-cold distilled water) for five minutes at room temperature. The reaction was initiated by addition of 400 μl of 0.5% potato starch solution (prepared in 20 mM phosphate buffer at pH 6.9, containing 6.7 mM sodium chloride) and incubated at 37 °C for 5 min. Final concentrations in each reaction mixture were 0.5 to 0.031 mg/ml of each plant extract, 1 U/ml of α-amylase and 0.25% (*w*/*v*) potato starch. The reaction was stopped by adding 100 μl of DNS reagent (96 mM 3,5-dinitrosalicyclic acid, 5.31 M sodium potassium tartrate in 2 M sodium hydroxide) and heating the mixture for 15 min at 85 °C in a water bath. After heating, 900 μl of distilled water was mixed with 100 μl aspirated from each sample mixture and absorbance read at 540 nm. The control mixtures were done in a similar manner except that the plant extract was replaced by 40 μl of DMSO or Acarbose (serial dilution) and with the blank; the enzyme solution was replaced by distilled water. Background readings were eliminated by subtracting the absorbance of the extract without substrate and enzyme from the absorbance of the extract and substrate mixture. The final results were expressed as IC_50_.

### Cell culture maintenance and differentiation

The C2C12 muscle and 3T3-L1 pre-adipocyte cells were provided by the Department of Biochemistry, Microbiology and Biotechnology, University of Limpopo. The cell lines were maintained in Dulbecco’s minimum-eagles medium (DMEM) (Hyclone, Thermo Scientific) supplemented with 10% (*v*/v) fetal bovine serum (FBS) and 1% (v/v) PSN (Penicillin: streptomycin: Neomycin) cocktail and incubated at 37 °C and 5% CO_2_ in a humidified incubator (Heracell 150i CO_2_ Incubator, Thermo Scientific). The culture medium was replaced with fresh medium every 2–3 days. C2C12 differentiation was induced by refreshing the growth medium (DMEM supplemented with 10% FBS and 1% PSN) with differentiation medium (DMEM supplemented with 2% horse serum and 1% PSN) for 4 days. The morphology of the C2C12 within this period changed to that of myotubules [21]. On the other hand replacing the growth medium with 3T3-L1 differentiation medium (0.5 mM IBMX, 1 μM dexamethasone, 5 μg/ml Insulin, DMEM, 10% FBS and 1% PSN) induced differentiation of the 3T3-L1 cells. On day 4, the differentiation medium was replaced with post differentiation medium (DMEM, Insulin, 10% FBS and 1% PSN) until day 8, with the medium changed every 2 days. By day 8, the pre-adipocytes were fully differentiated [22].

### Cytotoxicity assays

Toxic effects of each plant extract was determined by the 3-(4, 5-dimethylthiazol-2-yl)-2, 5-diphenyltetrazolium bromide (MTT) (Sigma Aldrich, SA) assay [23]. The C2C12 muscle and 3T3-L1 cells were seeded at a density of 5 × 10^3^ cells/well in 96 well plates and incubated at 37 °C and 5% CO_2_ overnight. Both cell lines were treated with various concentrations of each plant extract for 24 h. After 24 h of treatment, the treatment medium was aspirated carefully and replaced with 100 μl of MTT (1 mg/ml) in each well and further incubated for 3 h at 37 °C in the incubator. The formazan product was solubilized in 100 μl of DMSO the absorbance reading at 560 nm recorded using the Glomax microtiter plate reader (Promega, U.S.A). Untreated cells served as an experimental control from which the toxic effects of the extracts were determined. Results obtained were expressed as CC_50_ in μg/ml.

### Glucose uptake assay

The ability of the extracts to induce glucose uptake was determined using a published method [24]. Differentiated C2C12 and 3T3-L1 cells were treated with non-cytotoxic concentrations of the extracts for 3 h in a humidified incubator at 37 °C. After 3 h of incubation, 1 μl of each sample treatment was mixed with 99 μl of glucose perfect plus reagent (Audit Diagnostics, SA) and further incubated at 37 °C for 30 min. The absorbance was then read at 500 nm using the Glomax microtiter plate reader (Promega, U.S.A). The initial amounts of glucose present in each well were determined prior to incubation, just after treatment. Untreated cells served as a standard control and insulin (3000 mIU/ml) served as a positive control. The results obtained were expressed as % glucose uptake by the cells.

### GLUT4 translocation

The amount of GLUT4 translocated to the cell membrane was determined using immunocytochemistry [25]. Differentiated cells that induced high amounts of glucose uptake were chosen and seeded in 6 well plates at the density of 1.5 × 10^4^ cells/well on coverslips and incubated at 37 °C overnight. The medium was decanted and replaced with various treatments including the combination of insulin (3000 mIU/ml) and the plant extract. Untreated cells served as a standard control and insulin (3000 mIU/ml) as a positive control. The samples were incubated at 37 °C in a humidified incubator with 5% CO_2_ for 3 h. After 3 h of incubation, the treatment medium was decanted and the cells were washed once with 1 x phosphate buffered saline (PBS). The cells were then fixed with 80% ice-cold methanol for 10 min. This was followed by washing the cells once with 1 x PBS and blocking the non-specific binding with 0.5% bovine serum albumin (BSA) at room temperature for 30 min. The blocking buffer was decanted and the cells were washed twice with 1 x PBS and further incubated with primary rabbit anti-GLUT4 antibody (In 0.5% BSA blocking buffer) for 1 h. The cells were thereafter washed twice with 1 x PBS and incubated with goat-anti-rabbit FITC conjugated secondary antibodies (IgG) for 30 min and again washed with 1 x PBS and incubated with Wuchi stain (30 μg/ml) for 10 min. Finally, the cells were washed with 1 x PBS and mounted on slides and viewed using immunofluorescence microscope at 40X magnification.

### Dots blot analysis of MAPKs

Parallel determination of the relative levels of phosphorylation of Mitogen Activated Protein Kinases (MAPKs) was done using the protocol provided by the KIT (Human phosphor-MAP array kit) (R&D Systems). The differentiated C2C12 cells were treated for 3 h after which the cells were washed once with 1 x PBS. The cell lysates were obtained by solubilizing the cells in 300 μl lysis buffer, pipetting up and down and rocking at 4 °C for 30 min, then centrifuging at 13000 rpm and supernatant stored at −20 °C. The non-bindings sites on the membranes (provided by the kit) were blocked for 1 h. During this time, a concentration of 300 μg/ml of protein from each treatment was mixed with 20 μl of detection antibody cocktail and incubated for 1 h. After blocking the membranes for 1 h, they were incubated at 4 °C overnight with the mixture of sample/antibody. After incubation overnight, the membranes were placed into individual containers containing 20 ml of 1 x wash buffer and subsequently washed three times (10 min each) with 1 x wash buffer. After ensuring that the entire wash buffer was removed, the membranes were further incubated with streptavidin-HRP on a shaker for 30 min at room temperature. The membranes were thoroughly washed 3X after incubation and ensuring the entire wash buffer is dried up before adding the chemi reagent mix by blotting the edges on a paper towel. The chemi reagent mix was evenly distributed on the membranes and covered by plastic protectors, by pressing on the surface to remove all the bubbles. The plastic protector was replaced by plastic wraps and the antigen-antibody complex was then visualized by photo-detection using the Syne-Gene Image analyzer (Bio-Rad, S.A).

### Statistical analysis

Data was analysed using Graphpad Instat Statistical Software, version 3.0; the results were expressed as ± SEM of the mean, from triplicates of 3 to 4 independent experiments. Statistical evaluation of the results was determined using the one way analysis of variance (ANOVA), employing Turkey-Kramer multiple comparison test and the results were considered significant at *P* < 0.05.

## Results

### Phytochemical screening

Table 1 shows the phytochemical screening of the defatted and non-defatted extracts of *A. karroo*. The results shows the presence of phenols, flavonoids, tannins, saponins and cardiac glycosides and the absence of steroids, terpenoids and carbohydrates in the defatted and non-defatted extracts. However, low amounts of saponins were detected in the defatted extract as compared to the non-defatted extract.

**Table 1:** Qualitative phytochemical tests of *A. karroo* extracts

	Phenols	Flavonoids	Tannins	Terpenoids	Cardiac glycosides	carbohydrates	Steroids	Saponins
Non-defatted	++	++	++	_	++	_	_	++
Defatted	++	++	++	_	++	_	_	+

### Phenolic, antioxidant and α-amylase enzyme inhibition

Total amounts of phenols are shown to decrease with defatting of the plant material (0.432 ± 0.014 TAE/mg) as represented in Table 2. Also, the potential of ascorbic acid to scavenge DPPH free radicals was lower (SC_50_ 0.59 ± 0.003 mg/ml) than both extracts of *A. karroo* (*P* < 0.001), with the highest scavenging activity observed for the non-defatted extract (SC_50_ 0.37 ± 0.027 mg/ml) (Table 2). As opposed to DPPH free radical scavenging activity, the ability of ascorbic acid to reduce ferric ions to ferrous ions showed a greater potential (EC_50_ 1.06 ± 0.006 mg/ml) than both extracts, with the defatted extract having more potent activity (EC_50_ 1.13 ± 0.017 mg/ml) than the non-defatted. Furthermore, both extracts significantly (*P* < 0.001) reduced enzyme activity compared to the standard control acarbose (IC_50_ 109.8 ± 3.914 μg/ml), with the highest activity observed in the defatted extract (IC_50_ 30.2 ± 3.037 μg/ml) (Table 2).

**Table 2:** Total phenols, antioxidants and pancreatic α-amylase enzyme inhibition

	Phenols (TAE/mg)	DPPH scavenging activity SC_50_ (mg/ml)	Ferric Reducing power EC_50_ (mg/ml)	α-amylase enzyme inhibition IC_50_ (μg/ml)
Non-defatted	0.449 ± 0.025	0.37 ± 0.027***^a^	1.65 ± 0.023***^a^	39.3 ± 1.715***^a^
Defatted	0.432 ± 0.014	0.40 ± 0.012***^a^	1.13 ± 0.017^a^	30.2 ± 3.037***^a^
Ascorbic acid	_	0.59 ± 0.003	1.06 ± 0.006	_
Acarbose	_	_	_	109.8 ± 3.914

Data is expressed as ± SEM from averages of three to four independent repeats. Significant difference between the control and extracts and between the extracts was considered at *P* < 0.05. A superscript ^a^signifies significant difference between the extracts and the asterisk significant difference between the control and extracts

### Cytotoxicity

The defatted extract of *A. karroo* showed low toxicity effects in both C2C12 muscle (568.09 ± 5.029) and 3T3-L1 adipose cells (329.71 ± 10.0) following 24 h of treatment (Table 3.3). Toxic effects of both extracts were low in C2C12 muscle cells than 3T3-L1 adipose cells as shown in Table 3.

**Table 3:** Cytotoxic concentration killing 50% of cells

Cytotoxic concentration values (CC_50_ μg/ml)		
	C2C12 Muscle cells	3T3-L1 Adipose cells
Non-defatted	491.95 ± 2.309	312.74 ± 1.975
Defatted	568.09 ± 5.029	329.71 ± 10.0

Data is presented as ± SEM from three average determinations

### Glucose uptake

**Fig. 1:**
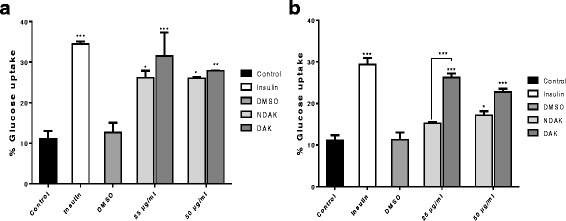
Glucose uptake by C2C12 muscle (**a**) and 3T3-L1 adipose cells (**b**) following 3 h treatment with extracts of *A. karroo*. Insulin at 3000 mIU/ml was used as a positive control. Untreated cells and DMSO were used as standard controls. NDAK = Non-defatted extract of *A. karroo*, DAK = Defatted extract of *A. karroo*. The results are presented as ± SEM from triplicate of three independent experiments. The statistical significance of the results was tested using One-way Analysis of Variance (ANOVA) employing the Turkey-Kramer multiple comparison test between the control and different groups and between groups. The results were considered significant at (*) *P* < 0.05, (**) *P* < 0.01 and (***) *P* < 0.001

Undifferentiated cell lines elicit poor glucose disposal, therefore, both cell lines were differentiated prior to treatment. The amounts of glucose taken up by differentiated C2C12 muscle (Fig. 1A) and 3 T3-L1 adipose (Fig. 1B) cells following 3 h of various treatments was measured and presented as % glucose uptake. Results obtained show that insulin resulted in the highest amounts of glucose uptake by C2C12 muscle (34.49% ± 0.599) and 3T3-L1 adipose cells (29.37% ± 1.559). Treatment with the defatted and non-defatted extracts at both 25 and 50 μg/ml resulted in significant glucose uptake by C2C12 muscle and 3T3-L1 adipose cells when compared to the untreated controls, except for the non-defatted extract at 25 μg/ml in 3T3-L1 adipose cells. The most active extract was the defatted extract at 25 μg/ml which shows an increase in glucose uptake by C2C12 muscle (31.52%) and 3 T3-L1 adipose cells (26.24%). Although glucose uptake induced by insulin was high above all treatments, there was no significant difference between insulin and both extracts at 25 and 50 μg/ml with both cell lines. The C2C12 muscle cells showed increased glucose uptake for all treatments compared to the 3 T3-L1 adipose cells. Furthermore, DMSO did not influence the amount of glucose taken up by both cell lines.

### GLUT4 translocation

**Fig. 2:**
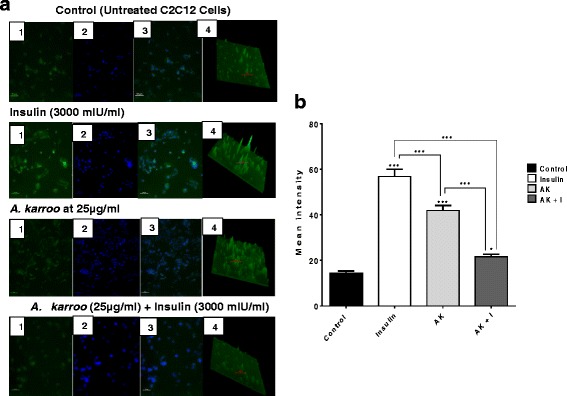
Fluorescence intensity (**a**) and mean intensity (**b**) following 3 h of treatment with insulin (positive control), *A. karroo* (AK) and insulin + *A. karroo* (AK + I) and untreated C2C12 cells (standard control). **a** 1 = green fluorescence represent FITC bound GLUT4 molecules, 2 = blue represent Wuchi stained nucleus, 3 = Overlay of images 1 and 2 and 4 = intensity surface plot. All the images were taken at a magnification of 40X. **b** The results obtained were expressed as means ± SEM. The statistical significance of the results was tested using one way ANOVA, employing the Tukey-Kramer multiple comparisons test. The *P* value significance was represented by an asterisk (*) for *P* < 0.05, two asterisks (**) for *P* < 0.01 and three asterisks (***) for *P* < 0.001

GLUT4 translocation in the C2C12 muscle cells was only assessed for the most active extract, in which case the defatted extract resulted in low toxicity and high enzyme inhibition and glucose uptake in both cell lines (particularly the C2C12 cells). Treatment of C2C12 cells with insulin, extract alone and treatment combination (extract + insulin) for 3 h show variations in the levels of GLUT4 translocation as seen both qualitatively (Fig. 2A) and quantitatively (Fig. 2B). GLUT4 translocation was significantly lower in the standard control than insulin (*P* < 0.001), extract (*P* < 0.001) and treatment combination (*P* < 0.05). Treatment of cells with insulin significantly induced GLUT4 translocation compared to the extract alone (*P* < 0.001) and treatment combination (*P* < 0.001), with the treatment combination being significantly lower than the extract alone (*P* < 0.001).

### MAPK phosphorylation levels

**Fig. 3:**
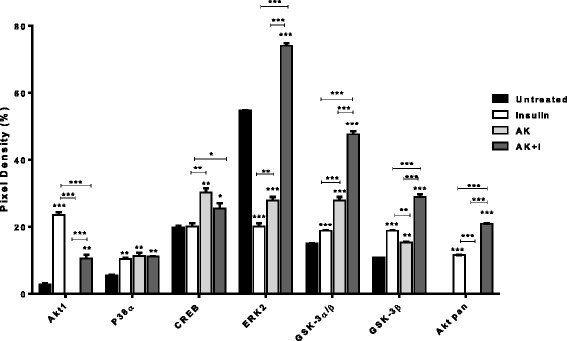
The C2C12 muscle cells were either untreated or treated with insulin (3000 mIU/ml), defatted extract *of A. karroo* (25 μg/ml) and a combination of the plant extract and insulin for 3 h. Data shown are from a four minute exposure to X-ray. The results obtained were expressed as means ± SEM. The statistical significance of the results was tested using one way ANOVA employing the Tukey-Kramer Multiple Comparisons Test. The P value significance was represented by an asterisk (*) for *P* < 0.05, two asterisks (**) for *P* < 0.01 and three asterisks (***) for *P* < 0.001

Figure 3 shows the phosphorylation levels of MAPKs associated with glucose metabolism following 3 h of treatment and results are presented as mean phosphorylation density. From the results obtained, insulin shows a significant increase in phosphorylation levels of Akt1 (*P* < 0.001) compared to the control, extract, and extract-insulin combination. Treatment with the plant extract alone completely suppressed phosphorylation of Akt1, while the treatment combination shows a significant reduction in the phosphorylation levels of Akt1. The control and plant extract did not show any phosphorylation of Akt pan (combination of Akt1, 2 and 3) as opposed to insulin and the treatment combination (insulin + extract), with the treatment combination showing significantly high phosphorylation levels (*P* < 0.001) among all treatment groups. Phosphorylation levels of P38α is shown to be significantly lower in the control compared to all treatment groups (*P* < 0.01), with no significant difference among treatment groups. A different trend was observed in the phosphorylation levels of CREB, where treatment with the extract alone and extract-insulin combination show significant phosphorylation levels as opposed to insulin, with no significant difference when compared to the control. Furthermore, treatment with the extract alone and extract-insulin combination show a significant increase in the phosphorylation levels of CREB compared to insulin alone. Treatment with insulin and extract alone show a significant reduction in the phosphorylation levels of ERK2 (*P* < 0.001) as opposed to the treatment with extract-insulin combination which show significantly high phosphorylation levels of ERK2 (*P* < 0.001) when compared to the control. Compared to the control, all treatment groups show a significant increase in the phosphorylation levels of both GSK-3α/β and GSK-3β; among all treatment groups, treatment with extract-insulin combination resulted in the most phosphorylation levels of GSK-3α/β and GSK-3β at *P* < 0.001.

## Discussion

Modulation of postprandial glucose levels through decreased glucose liberation from diet and glucose disposal by peripheral tissues such as muscle and adipose tissues are widely acceptable primary modalities for testing hypoglycaemic effects. In this study, we examined the phytochemical, phenolic and antioxidant constituent differences of the defatted and non-defatted extracts of *A. karroo*. Furthermore, biological activities that are determinants of antidiabetic effects were evaluated.

Medicinal plants contain diversity of phytochemicals that serve to protect them against pathogens. These phytochemicals have a proven history in the treatment of various diseases in humans and have served as a source for proven templates for drug development [26]. A review of available literature on antidiabetic effects of plants report that phytochemicals such as polyphenols, flavonoids, tannins, saponins, coumarins, anthraglycosides and terpenoids have the potential to induce antidiabetic effects [5, 26]. Our qualitative tests for the presence of phytochemicals showed the presence of phenols, tannins, saponins, flavonoids and cardiac glycosides in both defatted and non-defatted extracts and absence of terpenoids, carbohydrates and steroids. Presence of steroids [27] and carbohydrates in the extract were evaluated in the study because they can negatively affect the associated hypoglycaemic effects. Furthermore, defatting of the plant material did not influence the presence of inherent compounds.

Phenols are a diverse group of polar compounds attributed to the treatment and management of various conditions including; haemorrhagic shock, ageing, ischemia, Alzheimer, Parkinson’s disease, arthritis, gastrointestinal disorders, carcinogenesis, atherosclerosis and most importantly diabetes mellitus [28–30]. Following the determination of the qualitative presence of phenols in both extracts of *A. karroo*, the amounts of phenols contained in each extract were quantified. Defatting of the plant material influenced the outcome of the amounts of phenols, although the difference was not significant, the non-defatted extract had slightly higher amounts of phenols compared to the defatted extract. A previous study reported that the acetone leaf extract of *Vachellia karroo* (*Acacia karroo*) showed low amounts of phenols [31]. This is contrary to our finding in this study, where the acetone extract (non-defatted) is shown to have high amounts of phenols possibly due to geographical location, environmental factors, parasitic infestation or season of plant collection. On the other hand, we could not correlate the findings of the defatted extract with any previous study on the plant. In trying to understand the reasons behind the observed differences, we correlated our findings to findings on other plants other than *A. karroo*. A previous study [32] compared the phytochemical, antioxidant and bioactivity differences between the defatted and non-defatted extracts of various plants. Defatting of plant materials have been associated with yields containing high amounts of highly polarisable medium sized compounds, mostly phenols; which could be the exact reason why defatted extracts had high amounts of phenols. Hence we could attribute our findings to the nature of compounds present in the plant extract.

Phenols are considered as natural hosts of antioxidants. Antioxidants are molecules with the ability to prevent auto oxidation of bio-molecules and have also been greatly implicated in the prevention and progression of diabetes. The current findings in this study indicated that defatting of the plant material influenced the outcome of its antioxidant potential. The DPPH free radical scavenging potential was shown to decrease with defatting (0.40 ± 0.012), although the difference was not significant. Both the defatted and non-defatted extracts were shown to have very high electron donating abilities to quench DPPH free radicals when compared to ascorbic acid (0.59 ± 0.003) at *P* < 0.001. Contrary to the findings of DPPH free radical scavenging activity, the ferric reducing power was significantly (*P* < 0.001) improved with defatting (1.13 ± 0.017). Ascorbic acid had the high ability to reduce ferric ions Fe^3+^ to ferrous ions Fe^2+^ (1.06 ± 0.006) when compared to both extracts, with the non-defatted extract showing significantly low (*P* < 0.001) ferric reducing power (1.65 ± 0.023). This finding is consistent with a previous study [32] where the same trend was observed with other plants and was attributed to the removal of fats and oils (defatting). Adding to that, the nature of compounds present in the plant might have a direct bearing on the outcome, since phytochemicals produced in plants are a function of environment influences. However, looking at other phenolic compounds such as flavonoids and tannins might also give a clear picture to the outcome. Furthermore, the reducing power potential could be as a result of the number of phenol groups and position/number of hydroxyl groups and the scavenging activity could be attributed to the number of hydroxyl groups with redox properties [33].

Partial inhibition of carbohydrate digesting enzymes is one of the primary targets in the discovery of drugs with antidiabetic effects. These enzymes liberate glucose entering the blood circulation, thereby contributing to the levels of postprandial glucose [34, 35]. Hence, partial inhibition of these enzymes would result in the reduction of postprandial glucose levels. The extracts of *A. karroo* had high inhibitory effects on pancreatic α-amylase compared to acarbose, by more than 3 fold for the defatted and 2 fold for the non-defatted extracts. The observed inhibitory effects against α-amylase could be as a result of polyphenols [36, 37], in which case, antioxidants in this study could have also influenced the outcome of the inhibitory effects. Also, that defatting of the plant material yielded more compounds with inhibitory effects or unmasks their inhibitory potential against alpha amylase is a possibility. Furthermore, the interactive ability of proteins with phenols, perhaps due to the degree of hydroxylation and conjugation, could be the reason for the observed outcome. To the best of our knowledge, this is the first time this plant is reported to have α-amylase inhibitory effects.

Cytotoxic evaluation of the extracts showed the defatted and non-defatted extracts to be non-toxic against 3T3-L1 and C2C12 cells, with CC_50_ values >300 μg/ml. However, defatting of the plant material resulted in low toxicity effects against both cell lines. Furthermore, the toxic effects of the extracts were less on the C2C12 cells compared to the 3T3-L1 cells, possibly due to distinctive roles of the cell lines.

Apart from reducing enzyme activity, uptake of glucose from the circulation into peripheral tissues such as the liver, muscle and adipose tissue is the major process regulating blood glucose homeostasis [38]. It is widely accepted that adipose and muscle tissues are the major site of insulin-mediated glucose disposal due to their large contribution to body mass and serve as great modalities in diabetes studies. A majority of diabetics suffer a great loss of insulin resistance; hence compounds that mimic or increase insulin sensitivity are of major importance in the treatment of diabetes mellitus [7]. We examined the ability of the extracts of *A. karroo* to induce glucose disposal into the muscle and adipose cells. Results obtained revealed that both extracts have the potential of being a source of drug leads for treating diabetes mellitus; this is because both extracts increased glucose uptake at 25 and 50 μg/ml in both cell lines. Defatting of the plant material seemed to have improved the biological efficacy, probably as a consequence of the removal of interfering compounds. Also glucose uptake may differ from cell to cell as seen in our findings, where the C2C12 muscle cells improved glucose uptake as compared to the 3T3-L1 cells, which could be attributed to the distinct roles of different cells, as the compounds present in each extract may have distinctive effects on different cell lines. As to whether the observed glucose uptake is due to insulin mimicking/sensitising effects or other mechanisms is yet to be conclusively elucidated. Furthermore, we could as well not correlate our findings to any hypoglycaemic study on *A. karroo* because there are no reports on its hypoglycaemic potential, at least to the best of our knowledge. However, the capacity to which glucose was taken up in a period of 3 h is comparable to available studies on other plants with antidiabetic effects [8, 39].

Glucose disposal in peripheral tissues is mediated by insulin through special glucose transporters. Any defect in the translocation of these glucose transporters (most importantly GLUT4) from the intracellular vesicles to the surface membrane is associated with insulin resistance, thereby leading to hyperglycaemia. Glucose disposal in the muscle and adipose tissues is dependent upon GLUT4 translocation [40, 41]. In our study, we determined the major cause of the observed glucose uptake in the C2C12 muscle cells. The reason for this choice was the potential to induce high glucose uptake and low toxicity when treated with the extracts. Furthermore, the extract with overall best enzyme inhibition activity and glucose uptake was chosen; in this case, the defatted extract of *A. karroo* at 25 μg/ml. In this assay, a combination of insulin and the extract was included in order to ascertain whether the extract has additive or antagonistic effects when combined with insulin. The results obtained correlate with that of glucose uptake, suggesting that the glucose uptake observed was due to GLUT4 translocation. Furthermore, a combination of insulin and the extract showed antagonistic effects, suggesting the possibility of the extract’s ability to interact with one or more factors associated with insulin-mediated glucose transport signalling pathway, thereby blocking signal transduction caused by insulin. The possible role of chemicals, physical or biological alterations of factors involved in GLUT4 translocation can also not be ruled out. Despite the antagonistic effects observed with the treatment combination, this finding is beneficial since an extract that exerts such effects and equally promote GLUT4 translocation can serve as an alternative to insulin (insulin mimetic) treatment; and could be highly beneficial in the case of absolute absence of insulin and severe insulin resistance.

For GLUT4 molecules to translocate to the surface membrane, activation of the cascade involving various mitogen activated protein kinases and serine/threonine kinases augment to bring about desired effects. Having been certain that the observed glucose uptake was through GLUT4 translocation, we further assessed the modes of action by which the extract induce GLUT4 translocation. The phosphorylation profile of MAPKs [Akt1, Akt pan (Akt1, 2 and 3), p38α, CREB, ERK2, GSK3α/β and GSK3β] which are involved in glucose transport either directly or indirectly were examined using dot blot analysis.

The results obtained indicated that GLUT4 translocation indicated by insulin was through Akt1. Treatment combination of the defatted extract of *A. karroo* and insulin was shown to decrease the expression of Akt1, thus buttressing the notion that the plant extract in combination with insulin exert antagonistic effects, which could also be seen in the translocation of GLUT4 to the surface membrane. Contrary to insulin, treatment, the plant extract alone is shown to have completely suppressed the expression of Akt, which may suggest that the observed glucose uptake and GLUT4 translocation might not have been through Akt activation but rather through other pathways such as aPKCλ/ζ or p38α or proteins downstream Akt, although further studies need to be conducted in order to elucidate the exact mechanisms responsible for GLUT4 translocation. It was also evident that the defatted extract of *A. karroo* supresses all isoforms of Akt, as seen by the total suppression of Akt pan which is composed of all the three isoforms of Akt. The expression of Akt pan with insulin and the treatment combination could have been entirely through phosphorylation of Akt1 unlike Akt2 and Akt3. These findings are consistent with previous report [42], where compounds present within plants such as apigenin, kaemferol, quercetin and luteolin among a few, inhibit Akt-mediated glucose uptake, which might explain why Akt was completely suppressed and phosphorylation levels of Akt1 following treatment combination was reduced; this is also suggestive that the plant contains such compounds with the capabilities of inhibiting Akt. A study undertaken [43] demonstrated that some compounds such as garlic acid within plants inhibit GLUT4 translocation through suppressing Akt phosphorylation but stimulates GLUT4 translocation through aPKC ζ/λ phosphorylation.

Besides insulin as a stimulus, mitogenic or other stress stimulus activates p38α, resulting to glucose uptake via GLUT1 and GLUT4, protein synthesis and monitor cell survival [44, 45]. This study supports the claims that insulin activates p38α and could also account for the observed GLUT4 translocation among other functions; the phosphorylation levels of p38α due to insulin treatment was significantly higher than the control (*P* < 0.01). Surprisingly, the defatted extract of *A. karroo* was shown to slightly induce the phosphorylation of p38α than that of insulin and was also significantly different from the control (*P* < 0.01). This could suggest that the observed GLUT4 translocation induced by the defatted extract of *A. karroo* might be due to activation of proteins downstream of Akt (more especially the p38α) or other unknown pathways. Similarly, a treatment combination of insulin and extract also resulted in the phosphorylation of p38α slightly above that of insulin. Relating GLUT4 translocation induced by the extract alone and the treatment combination to phosphorylation levels of p38α may suggest that high phosphorylation levels of these treatments above that of insulin could be due to other stimuli such as compounds present in the extract. Furthermore, the possibility that p38α serves as the major regulator of GLUT4 translocation for the treatments, particularly the defatted extract of *A. karroo* cannot be ruled out.

Akt has also been associated with downstream phosphorylation of glycogen synthase kinases at positions Ser21 for alpha and Ser9 for beta respectively, thereby deactivating them. This enables glycogen synthase (GS) to remain active and promote glucose uptake and glycogen synthesis. Although both isoforms of GSK-3 have no direct bearing on GLUT4 translocation, they help in maintaining glucose regulation and cellular proliferative processes [46–48]. In this study, all the treatment groups exhibited significantly high phosphorylation levels of GSK-3β than the control. This suggests that there was increased glycogen synthesis from all treatment groups and that utilisation of energy for various metabolic processes was rather through a compensatory mechanism than the entire glucose taken up by the cells [49]. The results of GSK-3α/β phosphorylation were enhanced compared to GSK-3β with an exception of insulin, which suggests that insulin was more involved with phosphorylation of GSK-3β than GSK-3α. These results confirm that the plant extract has the ability to phosphorylate proteins downstream of Akt. Dysregulation of GSK-3 results in insulin resistance as observed in diabetic patients; which could possibly make the extracts beneficial in this aspect.

The cAMP response element binding protein (CREB) is one of the major important proteins that mediate nuclear transcription of various factors depending on the stimuli. The CREB is commonly known to be responsible for adaptation, cell survival, differentiation and proliferation [50, 51]. CREB can be activated by upstream proteins such as Akt, PKA, PKC, MSK-1, p90RSK and CAMK, each with a desired transcriptional response [50, 52, 53]. It was not surprising in this study that all treatment groups showed phosphorylation of CREB, even though only the extract and treatment combination of extract and insulin were significantly higher than the control at *P* < 0.01 and *P* < 0.05, respectively. These results indicate that there was transcription of various proteins responsible for executing desired functions based on the stimuli. Elevated levels in the extract and treatment combination could suggest increased protein transcription probably due to the fact that plants contain an array of compounds which may act as stimulus for transcription of different proteins and as such, the observed differences. Similar to the function of CREB, another protein called ERK-2 has also been associated with transcription of proteins and is involved in differentiation, cell proliferation, migration, adhesion and survival [54, 55]. Phosphorylation of ERK-2 due to the defatted extract of *A. karroo* and insulin was significantly lower than the control (*P* < 0.001) and higher in the treatment combination than the control (*P* < 0.001). It is not clear as to why the control exhibited very high phosphorylation levels of ERK-2, although it could be suggested that a recovery from starvation might have played a role in various processes which could be compensatory to the cells. Also, slow glucose uptake seems to have played a role in the untreated control relative to insulin and the extract alone. Very high phosphorylation levels of the treatment combination might suggest that there was no interference between the extract and insulin resulting to an additive response. As to whether this might have played a role in the reduction of glucose uptake requires further investigation.

## Conclusion

Both extracts of *A. karroo* possess hypoglycaemic potential and require further investigation, most importantly in in vivo studies. In addition to that, the defatted extract has a great potential of providing drug leads for the treatment of diabetes mellitus and the exact mechanism (s) responsible for the observed biological activity needs further elucidation. Studies are ongoing aimed at the isolation of compounds and/or the identification of sub fractions that can elicit the observed effects.

## Abbreviations

Akt: Alpha serine/threonine kinase
aPKCλ/ζ: Protein kinase lamda and zeta
CC_50_: Cytotoxic concentration exhibiting 50% activity
CREB: cAMP response element binding protein
DPPH: 2, 2-diphenyl-1-picrylhydrazyl
EC_50_: Concentration exhibiting 50% activity
ERK2: Extracellular signal regulated kinase
GSK-3α/β: Glycogen synthase kinase-3 alpha and beta
IC_50_: 50% Inhibitory concentration
MAPK: Mitogen activated protein kinases
P38: p38α mitogen-activated protein kinase
SC_50_: 50% Scavenging concentration
TAE: Tannic acid equivalence
